# Mechanism of action of *Panax notoginoside* against lung cancer in mice based on response to *CTSB* gene

**DOI:** 10.1186/s12906-020-03159-0

**Published:** 2020-11-25

**Authors:** Jizhou Zhang, Bin Zhou, Song Jin, Zhiyou Huang, Bidong Ma, Qiqi Shao, Wenzong Zhu

**Affiliations:** 1 Department of Oncology, Wenzhou Hospital of Traditional Chinese Medicine Affiliated to Zhejiang Chinese Medicine University, Wenzhou, China; 2 Nanjing University of Chinese Medicine, Nanjing, China; 3 Department of Nursing, Central Health Center of Zeya Town, Ouhai District, Wenzhou, China; 4 Internal Medicine of Traditional Chinese Medicine, Wenzhou Hospital of Traditional Chinese Medicine Affiliated to Zhejiang Chinese Medicine University, No. 9 Jiaowei Road, Lucheng District, Wenzhou, China

**Keywords:** *CTSB* gene, Inhibitory effect, Lung cancer, *Panax notoginoside*, Proliferation

## Abstract

**Background:**

This study aimed to investigate the mechanism of action of *Panax notoginoside* (PNS) against lung cancer and inhibition of lung cancer cell proliferation by the drug at different concentrations in a mouse model, considering the cathepsin B (*CTSB*) gene as a target.

**Methods:**

The mice were randomly assigned into the following five groups: normal control, tumor-bearing, low-dose *Panax notoginoside* (TSPN), medium-dose TSPN, and high-dose TSPN. All mice were treated with physiological saline or TSPN at different concentrations for 28 days consecutively by gavage. The tumor size was measured, the tumor growth was observed, and the survival curve was drawn. At different time points, the expression of the *CTSB* gene was detected using quantitative fluorescent polymerase chain reaction, Western blot analysis, and indirect immunofluorescence. The serum indices, such as carcinoembryonic antigen (CEA), neuron-specific enolase (NSE), and Soluble fragment of cytokeratin 19 (CYFRA21), were detected by enzyme-linked immunosorbent assay.

**Results:**

In vivo, PNS could directly inhibit the expression of the *CTSB* gene in tumors of mice, limit tumor growth, and alter tumor-related indices, such as CEA, NSE, and CYFRA21 levels, in the serum to different extents simultaneously.

**Conclusion:**

*CTSB* gene was closely related to the pathogenesis of lung cancer. PNS could act on the *CTSB* gene, downregulate the expression of *CTSB* in lung cancer cells, inhibit the proliferation and invasion of tumors, and prolong the survival period.

**Supplementary Information:**

The online version contains supplementary material available at 10.1186/s12906-020-03159-0.

## Background

Kaur [1] indicated an increase in knowledge on the role of phytodaptogens in moderating biological and molecular processes against carcinogenic cells with a prospect of fascinating possibilities. The well-known adaptogens and immunomodulators, such as *Rhodiola rosea* and *Panax notoginseng*, have been shown to have significant antioxidant and anticarcinogenic properties due to the presence of various biologically active chemical compounds. This strongly supported the view that the beneficial properties of plant adaptogens should be contemplated as an adjuvant because they hold immense potential in the fight against cancer with their capability in orchestrating molecular mechanisms in restoring homeostasis in the body system.

*Panax notoginoside* (PNS) is the main active ingredient in traditional Chinese medicine, Notoginseng, with a content of more than 13%. It has a wide-range pharmacological action, and it is the main component with medicinal value. It contains mainly monomer saponins, such as ginsenoside Rb1, ginsenoside Rg1, ginsenoside Rh1, notoginsenoside R1, and ginsenoside Re [2]. It possesses strong anti-tumor activity, enhances learning and memory in mice, causes hemostasis, and exerts a blood-activating dual-directional regulatory effect [3]. At present, many scientists believe that Notoginseng possesses important anti-tumor effects. Investigations were performed at the molecular level, and Notoginseng was widely applied in treating lung cancer in clinic. However, the mechanism of action of Notoginseng against lung cancer and inhibition of the proliferation of lung cancer cells by the drug still remain unclear. Whether the ingredient PNS (active component group) in Notoginseng has a significant role in lung cancer is still unclear. Some previous studies reported that the cathepsin B (*CTSB*) gene was highly expressed in gastric cancer, esophageal cancer, glioma, prostate cancer, and breast cancer tissues, and the expression of the *CTSB* gene was upregulated at the gene and protein levels [4–9]. The CTSB gene may participate in the development and progression of tumors and is crucial in initiating apoptosis. Multiple studies proved that the elevated expression level of the *CTSB* gene was related to the progression of cancer. Therefore, *CTSB* was considered as a potential therapeutic target for tumors [10]. In this study, PNS, an active component group of Notoginseng, was used as an intervention drug and the *CTSB* gene as a target. The molecular mechanism underlying the anti-tumor activity and inhibition of the proliferation of lung cancer cells by the drug was investigated by treating the mice having lung cancer with different doses of the drug, thus providing an experimental basis for the clinical treatment of lung cancer using Notoginseng.

## Methods

### Materials

Xuesaitong granules (Luotai) (pharmaceutical standard of the Ministry of Health of the People’s Republic of China) were selected, taking PNS as a raw material. The product was prepared by adding appropriate ingredients [11]. The drug was stable and easy to use with less component interference. The granules (Luotai) were manufactured by Xuesaitong Pharmaceutical Co. Ltd. (Kunyao group; China Food and Drug Administration approval number Z20113072, 1.5 g/bag, equivalent to 50 mg of PNS without sucrose).

### Experimental animals

Eighty BALB/c nude specific-pathogen-free (SPF) mice (of either sex, aged 6–8 weeks, weighing 18–25 g) were purchased from Shanghai SLAC Laboratory Animal Co., Ltd. (license number: SCXK) (Table 1). They were raised in the SPF environment at a temperature of 20 °C–25 °C under a light/dark cycle of 12 h/12 h. Drinking and sleep were unconstrained.

**Table 1:** Animal grouping information

Group	Age (week)	Weight (Gram)	Sex (number)	
			Female	Male
Normal control	7.009 ± 0.625	21.942 ± 2.091	11	5
Tumor bearing	6.95 ± 0.448	22.029 ± 2.409	10	6
Low-dose TSPN	7.044 ± 0.535	21.592 ± 1.966	5	11
Medium-dose TSPN	7.093 ± 0.523	21.603 ± 2.023	9	7
High-dose TSPN	6.946 ± 0.512	22.201 ± 1.946	5	11

*TSPN Panax notoginoside* treatment

### Reagents

The protein extraction kit, protein quantitative polymerase chain reaction (PCR) kit, HRP-labeled goat anti-mouse immunoglobulin G (IgG), and HRP-labeled goat anti-rabbit IgG were purchased from Beyotime Biotechnology (Songjiang, Shanghai). The CTSB and caspase-3 antibodies were provided by Abcam (Pudong, Shanghai). An SYBR Green PCR kit was obtained from Thermo Fisher (USA). Dulbecco’s modified Eagle’s medium (10569044) and fetal bovine serum (10100154) were purchased from Invitrogen. Streptomycin mixing solution (100×) and trypsin–ethylene diamine tetraacetic acid (EDTA) digestion solution (0.25%) were provided by Solarbio (Beijing, China). Carcinoembryonic antigen (CEA) enzyme-linked immunosorbent assay (ELISA) kit, neuron-specific enolase (NSE) ELISA kit, and CYFRA21-1 ELISA kit were purchased from Iiyinmei (Wuhan, China).

### Establishment and grouping of the animal model

A549 cells were cultured using the conventional method and resuspended in 0.1 mL of phosphate-buffered saline at a concentration of 2 × 10^7^ cells for each mouse. Human alveolar basal epithelial cells of lung adenocarcinoma A549 were transplanted into the right forearm underarm of mice using a No. 20 trocar to construct the model. Eighty mice were randomly assigned into five groups (*n* = 16): normal control, tumor bearing, low-dose TSPN, medium-dose TSPN, and high-dose TSPN. The control group mice were not inoculated with tumor cells; they were treated with 0.4 mL of physiological saline by gavage. The remaining 64 mice were subcutaneously injected with A549 cells after sterilization of the skin under the right forearm underarm. The mice in the tumor-bearing group were treated with 0.4 mL of physiological saline daily by gavage. The mice in the low-dose group were treated with TSPN at a dose of 50 mg/(kg · day) by gavage daily (0.4 mL). The mice in the medium-dose group were treated with TSPN at a dose of 100 mg/(kg · day) by gavage daily (0.4 mL). The mice in the high-dose group were treated with TSPN at a dose of 200 mg/(kg · day) by gavage daily (0.4 mL). All mice were treated with physiological saline or TSPN at different concentrations for 28 days consecutively [12]. The tumor size was measured during this period, and the tumor growth was observed. On days 0, 14, and 28 of treatment, the blood samples were collected via the caudal vein. CEA, NSE, and CYFRA21-1 levels were measured by ELISA.

### Calculation of survival time and drawing of the survival curve

Survival status, tumor growth, tumor formation time, and death time of mice were observed from the day of tumor cell inoculation to day 28 to calculate the median survival time and draw the survival curve. On day 28, half of the mice were sacrificed. The remaining mice in all groups, except the control group, were continuously raised until natural death. The death times were observed and recorded to calculate the overall survival and draw the curve.

### Detection of serum CEA, NSE, and CYFRA21-1 levels

Serum CEA, NSE, and CYFRA21-1 levels were measured using a double-antibody sandwich ELISA kit (Jiyinmei, Wuhan, China) following the manufacturer’s protocol. The tumor markers from the blood samples of mice in each group were measured on days 0, 14, and 28. The changes in cancer cells before and after the intervention and during the experiment were real-time monitored and recorded to evaluate the outcome after the intervention.

### Quantitative fluorescent PCR detection

On day 28, half of the mice were sacrificed, and the tumors were removed to extract total RNA and protein, followed by detection using quantitative fluorescent PCR. The primer used in the PCR experiment referred to relevant sequences in the National Center for Biotechnology Information (NCBI) database, designed using Oligo 7 software and confirmed by BLAST comparison (Table 2). The siRNA targeting the *CTSB* gene was designed online using BLOCK-iT RNAi Designer software. The primer and siRNA were synthesized by General Biosystems (Anhui) Corporation Limited, and the instructions were strictly followed.

**Table 2:** Primer and siRNA sequences

Gene	Primer sequence	Amplified fragment size (bp)	Location
Cathepsin B	Upstream primer: 5′-TACAATTCCTACAGCGTCTCC-3′ Upstream primer: 5′-GTGCCATTCTCCACTCCC-3′	194	679–872
Caspase-3	Upstream primer: 5′-CATGGAAGCGAATCAATGGACT-3′ Upstream primer: 5′-CTGTACCAGACCGAGATGTCA-3′	139	64–202
GAPDH	Upstream primer: 5′-AATCCCATCACCATCTTC-3′ Upstream primer: 5′-AGGCTGTTGTCATACTTC-3′	218	436–653
siRNA-NC	5′-GUACGCCAAAAGUUAAACC-3′		
siRNA1	5′-CCUGUCGGAUGAGCUGGUCAACUAU-3′		
siRNA2	5′-CCAGUACCUCCAAGCAAGUAGCUUU-3′		
siRNA3	5′-UGCAUCUAUCGAGUUUGCAAUGUCA-3′		

### Western blot analysis

On day 28, half of the mice were sacrificed to remove the tumor. The expression level of the *CTSB* gene in some tumors was detected by Western blot analysis, strictly following the instructions.

### Indirect immunofluorescence detection

On day 28, half of the mice were sacrificed and the tumors were removed. Some tumors were fixed with formaldehyde and embedded in paraffin. The expression level of the *CTSB* gene in the tumor was observed by immunofluorescence, strictly following the instructions.

### Statistical analysis

The results were expressed as mean ± standard deviation and analyzed using Prism 5.0 software (GraphPad, CA, USA). The Student *t* test and one-way analysis of variance were used to analyze the difference between groups. A *P* value < 0.0 indicated a significant difference.

## Results

### TSPN inhibited the proliferation of xenograft tumor in nude mice

**Fig. 1:**
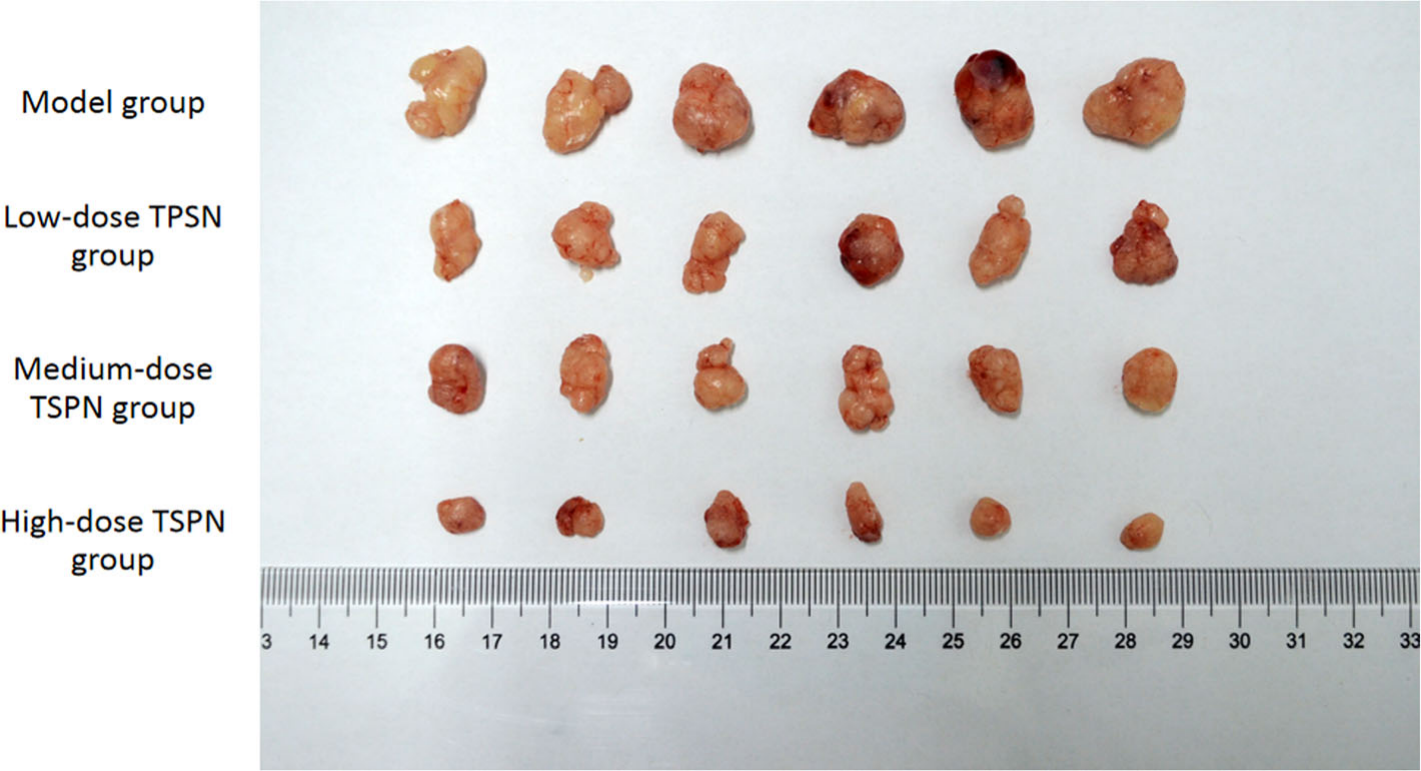
Tumor size in different groups after 28 days of TSPN treatment

**Fig. 2:**
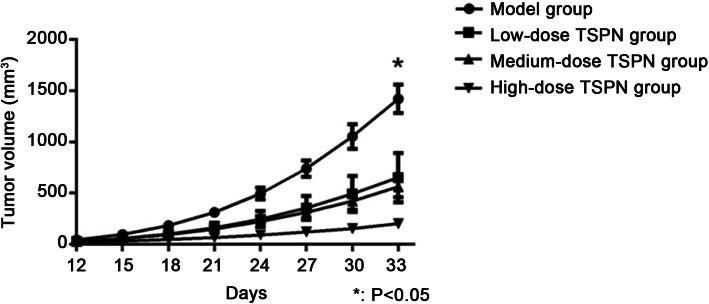
Tumor growth curve in different groups after 28 days of TSPN treatment

After 28 days of TSPN gavage treatment in mice inoculated with A549 cells, the tumor growth rate significantly slowed down in a dose-dependent manner in the TSPN treatment groups compared with the control group. A significant change in tumor size was observed in different groups (*P* < 0.05) (Figs. 1 and 2).

### TSPN inhibited the expression of CTSB mRNA in the tumor

**Fig. 3:**
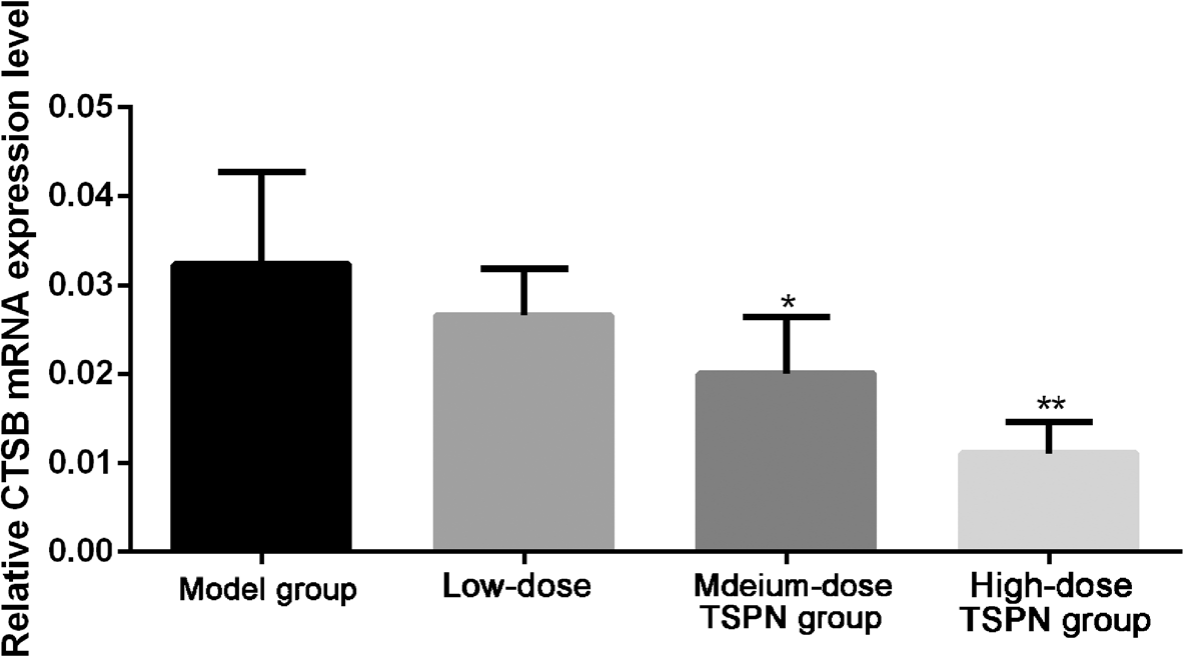
Expression levels of CTSB mRNA in different groups after 28 days of TSPN treatment. ^*^P < 0.05, ^**^P < 0.01

After injecting A549 cells subcutaneously, the mice were treated with three different doses of TSPN by gavage. After 28 days, the tumors from different groups were taken out. The result of quantitative fluorescent PCR indicated that the expression level of CTSB mRNA reduced after TSPN gavage treatment compared with that in the tumor-bearing group, and was more significant with an increase in the dose (Fig. 3).

### TSPN inhibited the expression of CTSB protein in the tumor

**Fig. 4:**
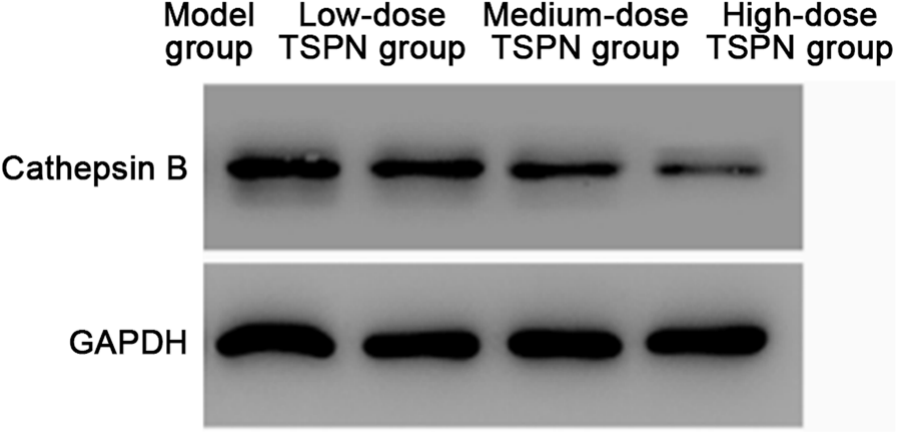
Expression levels of CTSB protein in different groups after 28 days of TSPN treatment. ^*^P < 0.05, ^**^P < 0.01

**Fig. 5:**
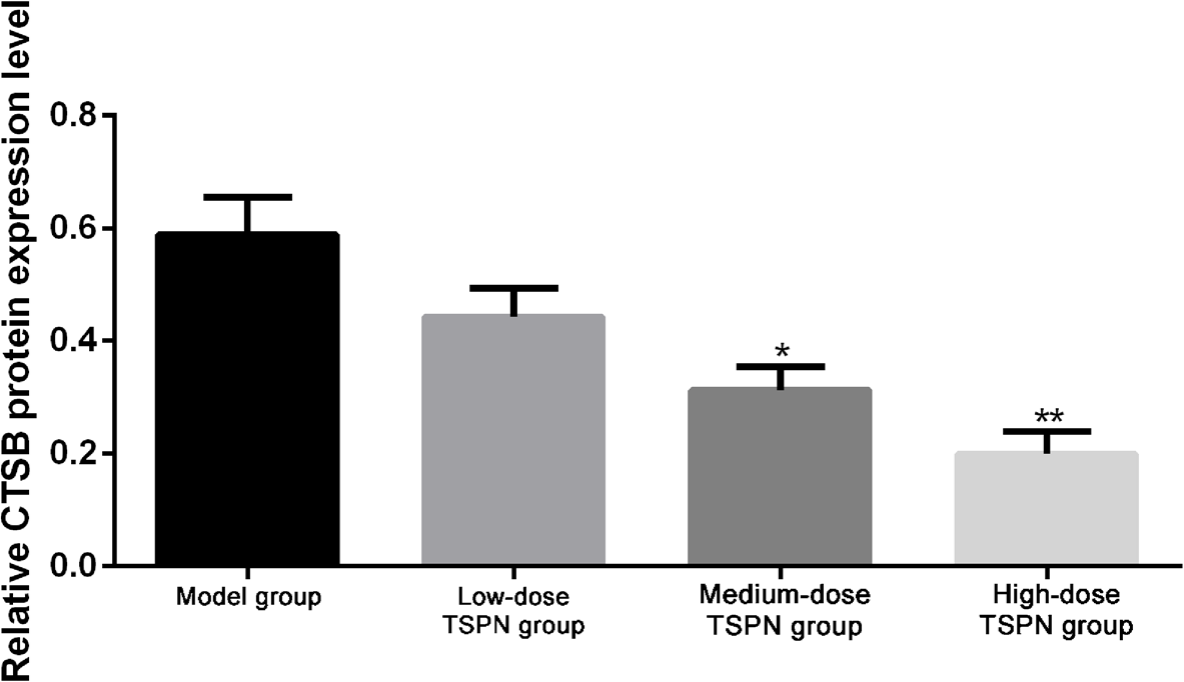
Comparison of the gray value of CTSB protein in tumors between different groups on day 28 of TSPN treatment detected by Western blot analysis. ^*^P < 0.05, **P < 0.01

**Fig. 6:**
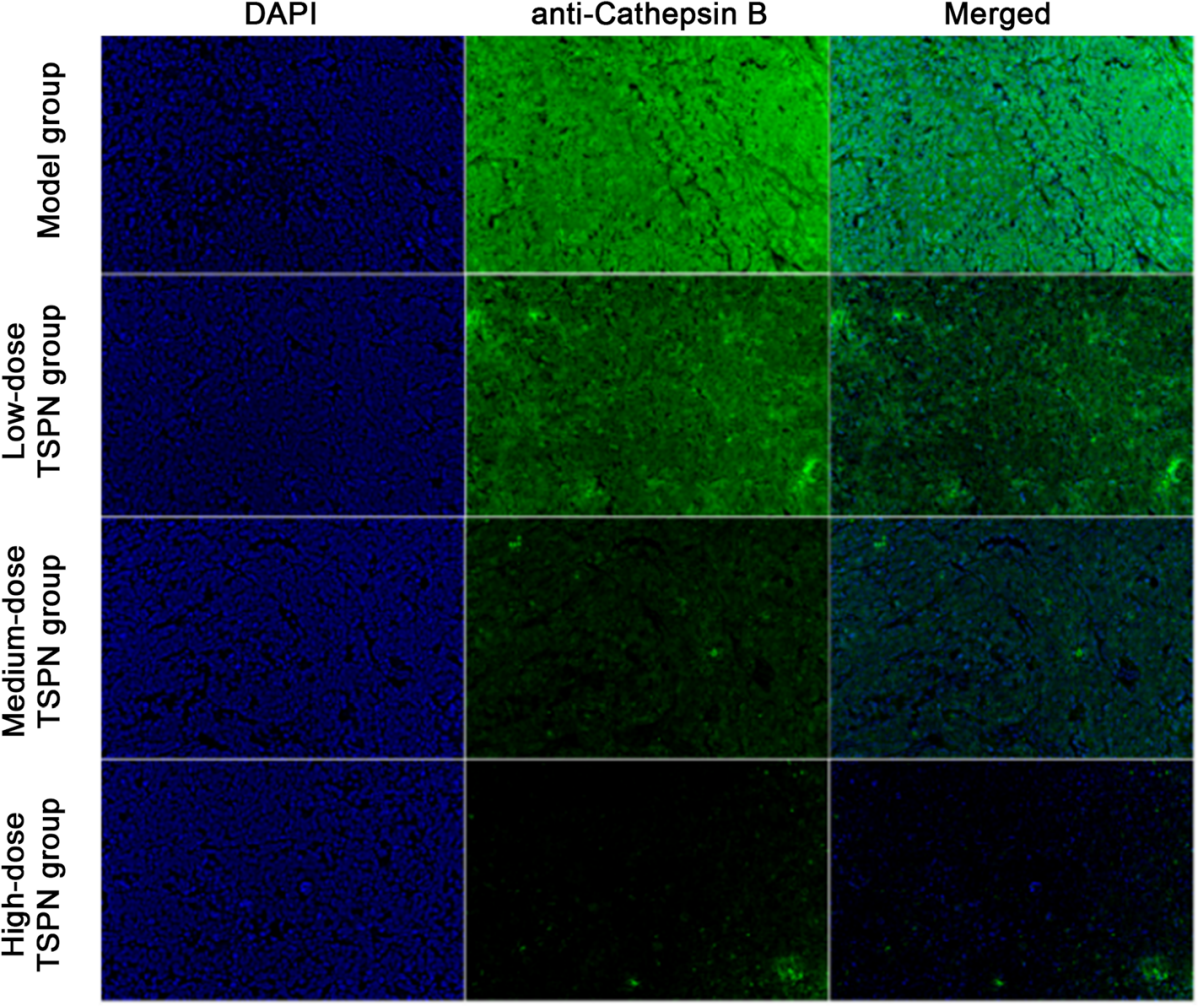
Expression levels of CTSB protein in different groups detected by indirect immunofluorescence

After subcutaneous injection with A549 cells, the mice were treated with three different doses of TSPN by gavage. After 28 days, the tumors in different groups were removed. The results of Western blot analysis and indirect immunofluorescence indicated that the expression of CTSB protein reduced after TSPN gavage treatment in a dose-dependent manner compared with the tumor-bearing group (Figs. 4, 5 and 6).

### TSPN reduced the CEA, NSE, and CYFRA21 levels in peripheral blood after inoculation with A549 cells

**Fig. 7:**
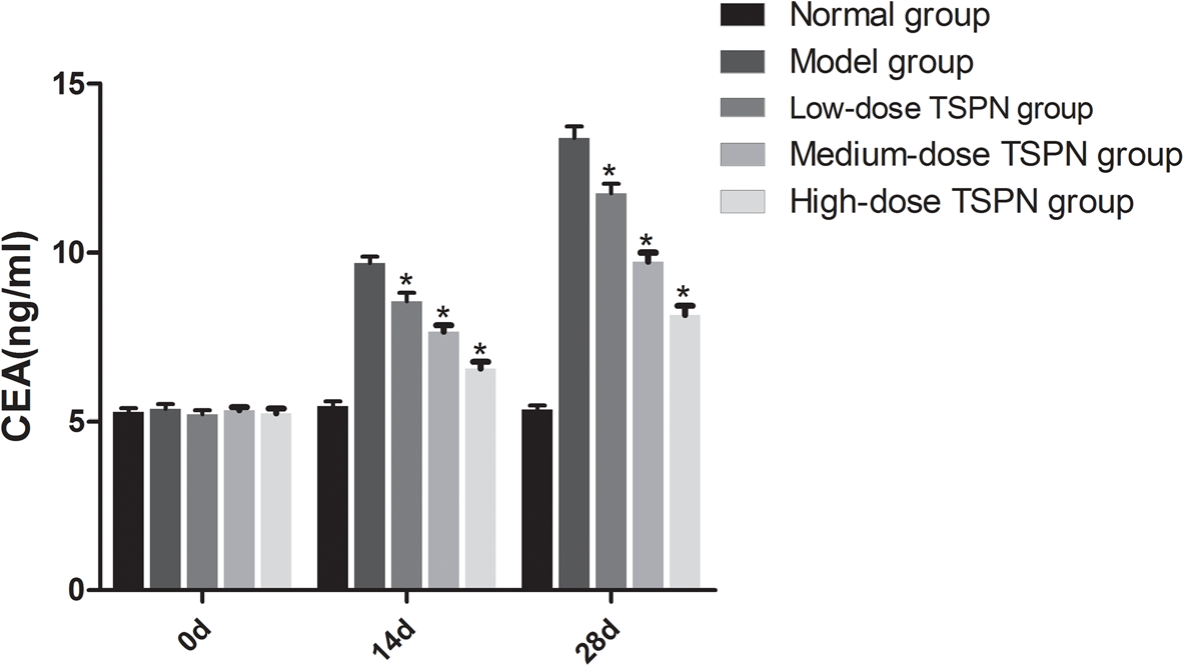
Change in the CEA level in peripheral blood on days 0, 14, and 28 of TSPN gavage treatment (^*^P < 0.05)

**Fig. 8:**
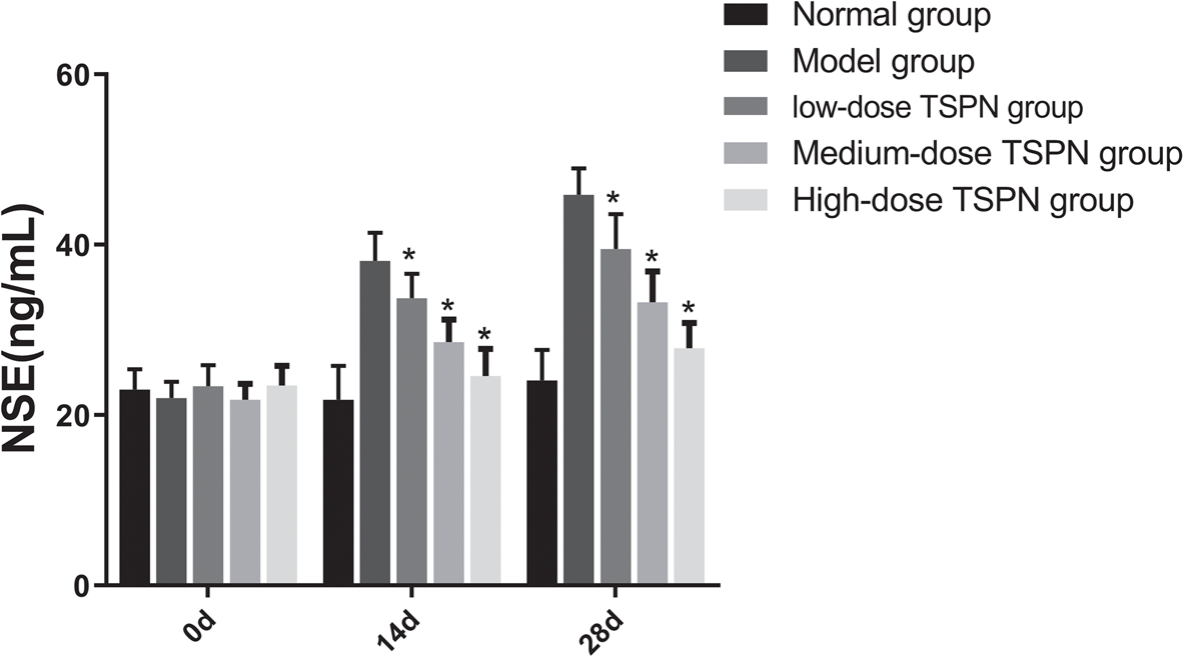
Change in the NSE level in peripheral blood on days 0, 14, and 28 of TSPN gavage treatment (^*^P < 0.05)

**Fig. 9:**
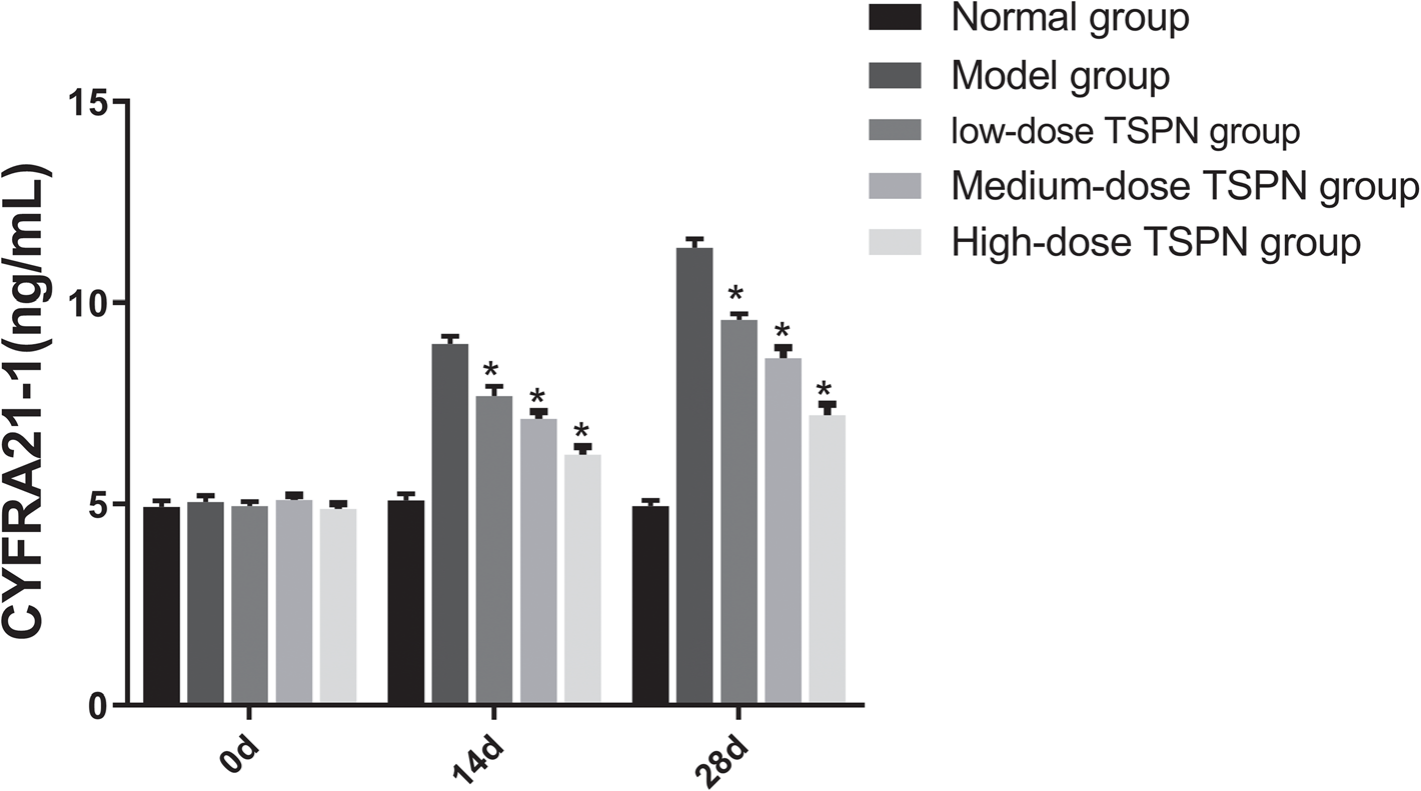
Change in the CYFRA21 level in peripheral blood on days 0, 14, and 28 of TSPN gavage treatment (^*^P < 0.05)

The nude mice were subcutaneously inoculated with A549 cells and treated with three doses of TSPN by gavage for 28 days. The CEA, NSE, and CYFEA21 levels in the peripheral blood were detected on days 0, 14, and 28. The result indicated that after inoculation with A549 cells, the CEA, NSE, and CYFEA21 levels in the peripheral blood gradually increased. After TSPN gavage treatment, the levels gradually decreased in a dose-dependent manner (Figs. 7, 8 and 9).

### TSPN treatment extended the survival time of tumor-bearing mice

**Fig. 10:**
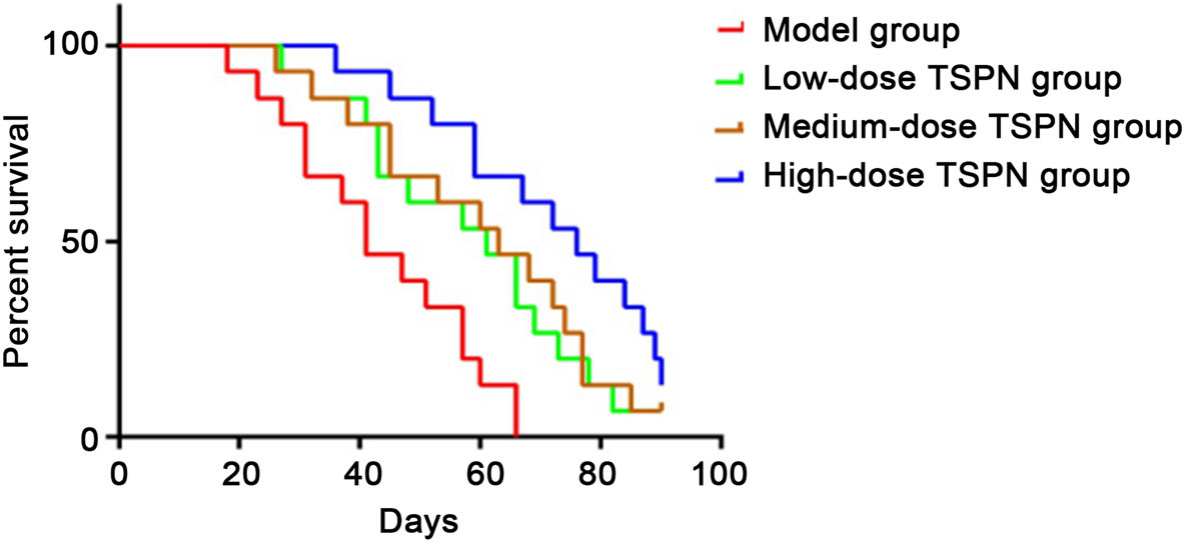
Survival curve of each group on day 28 of TSPN gavage treatment at different doses

The nude mice were subcutaneously inoculated with A549 cells and raised until day 90. All mice in the control group survived on day 66, while one each survived in the TSPN low-dose and medium-dose groups on day 90. Two survived in the high-dose group on day 90. The survival times in the three TSPN treatment groups were significantly longer than those in the control group (*P* = 0.025, *P* = 0.021, and *P* = 0.005, respectively). Besides, the survival time in the high-dose group was significantly longer than that in the low-dose and medium-dose groups (*P* = 0.034 and *P* = 0.042, respectively) (Fig. 10).

## Discussion

Cancer is still one of the main threats to human health. The incidence and morbidity in China have increased every year [13]. More than one sixth of deaths caused by cancer are attributed to lung cancer [14]. The therapy for lung cancer is mainly modern medicine, including early surgical treatment, chemotherapy, radiotherapy, and immune-targeted therapy [15]. However, recent therapies still have some defects. For example, chemoradiotherapy causes reduced immunity in patients, the target drugs are expensive, the drug resistance exists, and the individual difference in efficacy is large. The current clinical information has indicated that the use of traditional Chinese medicine helps improve the immunity of patients with cancer, stabilize tumor foci, and improve clinical manifestations [16]. Kaur mentioned that *Panax notoginseng* contained a variety of bioactive compounds, which could promote immune activity by regulating T-cell immune biochemical factors, transcription factors, some genes, and factors related to tumor development and progression, and had significant antioxidant and anticancer effects. Traditional Chinese and Western medicines for treating lung cancer have gained increasing attention [1].

In the pathogenesis of lung cancer, the most common is non-small-cell lung cancer; the incidence rate of lung adenocarcinoma is the highest. Progress in the treatment of lung adenocarcinoma is of great significance in the treatment of lung cancer. Therefore, this study also focused on lung adenocarcinoma. A549 cell line is a common cell line of human lung adenocarcinoma, also called adenocarcinoma human alveolar basal epithelial cell. It was first developed in 1972 by D. J. Giard et al. Through an explant tumor of a 58-year-old white male patient, the lung tumor tissue was transferred and cultured [17]. A549 cells can spread some substances, such as water and electrolytes, through the alveoli. At the same time, they can also synthesize lecithin and contain highly unsaturated fatty acids, which are important for maintaining the cell membrane phospholipids. A549 cells are widely used in the type II lung epithelial cell model. As an in vitro model of drug metabolism, A549 cells can also be used as a transfection host in many lung cancer studies. Therefore, A549 cells were selected in the present study. The feasibility ratio of A549 cells was confirmed to be higher in the preliminary experiment.

CTSB is one of the members of the cysteine superfamily found to be upregulated in multiple tumors. In a previous study, the downregulation of the expression of the *CTSB* gene in different tumors by antisense RNA, siRNA, and shRNA technique suggested that the downregulation of the *CTSB* gene was related to the development and progression of tumors. Rao et al. comprehensively analyzed the role of the downregulation of the *CTSB* gene or other members of proteolytic enzyme pathways in the brain tumor. The result again indicated that the downregulation of *CTSB* was related to the proliferation and invasion of tumors [18]. In a mouse breast cancer model, the downregulation of shRNA in the expression of the *CTSB* gene reduced the degradation of type I collagen and inhibited bone metastasis [19]. In meninges, the downregulation of *CTSB* and matrix metalloprotein-9 inhibited the proliferation of tumors and reduced tumor cell proliferation, invasion, angiogenesis, and downstream kinase signaling pathway activation [20]. Thus, the aforementioned findings confirmed that the upregulation of the expression of the *CTSB* gene promoted the malignant phenotype of the tumor. The role of the *CTSB* gene in tumor development and progression (initiation, proliferation, angiogenesis, invasion, inflammation, apoptosis, and metastasis) was comprehensively investigated using a mouse model.

A previous study found that the expression of *CTSB* significantly reduced after using TSPN at different concentrations to treat lung cancer A549 cells, suggesting TSPN in A549 cells could promote apoptosis and inhibit cell proliferation via inhibiting the expression of the *CTSB* gene. After injecting A549 cells subcutaneously, the mice were treated with different doses of TSPN by gavage. After 28 days, the mice were sacrificed, and tumors in different groups were removed. The result of quantitative fluorescent PCR indicated that compared with the tumor-bearing group, the expression of the *CTSB* gene after TSPN gavage treatment reduced and was more significant with an increase in the dose. Compared with the control group, the tumor growth rate in the TSPN treatment group was significantly lower and more significant with the increase in the dose.

The role of the *CTSB* gene in the development and progression of tumors still remains unclear. A preclinical study of the tumor model in mice indicated that broad-spectrum inhibitors targeting CTSB and other cysteine cathepsins could effectively inhibit the proliferation of tumors. This study found that TSPN inhibited the expression of the *CTSB* gene and controlled the proliferation of tumors in lung cancer cells and the xenograft tumor model in mice. Meanwhile, it could also significantly improve the survival time of tumor-bearing mice. The serum levels of CEA, NSE, and CYFRA21 on days 0, 14, and 28 revealed that the levels in the tumor-bearing group gradually increased with time. However, the serum CEA, NSE, and CYFRA21 levels in the TSPN gavage treatment groups reduced, and the extent of reduction had a positive correlation with the TSPN concentration. This suggested that TSPN exerted an anti-tumor effect by downregulating the expression of the *CTSB* gene.

This study had some limitations. The optimal effective concentration of TSPN to downregulate the expression of *CTSB* is still unclear. For example, three effective concentrations, 50 mg/kg · day, 100 mg/kg · day, and 200 mg/kg · day were used in this experiment. The results showed that the effects of these three concentrations were dose dependent; 200 mg/kg · day was more advantageous in this experiment. However, 200 mg/kg · day was not necessarily the best effective concentration of TSPN; 250 mg/kg · day might be more advantageous than 200 mg/kg · day. This problem should be further verified in the follow-up studies. Future studies should explore the optimal effective concentration of TSPN.

## Conclusions

The *CTSB* gene was closely related to the occurrence and development of lung adenocarcinoma. CTSB protein and CTSB mRNA were highly expressed in lung cancer. The expression level was directly proportional to the malignant degree of lung cancer. The higher the expression level, the faster the growth and progression of lung cancer. At the same time, *Panax notoginseng* saponins were used for the intervention experiment. The results showed that PNS could effectively act on the *CTSB* gene so that CTSB protein and CTSB mRNA could be induced. The downregulation of mRNA expression limited the growth of tumors and altered tumor-related indexes (such as CEA, NSE, and CYFRA21) to varying degrees. This indicated that PNS might inhibit the proliferation and invasion of the tumor through the *CTSB* gene and prolong the survival time of mice with lung cancer, providing an experimental basis for treating lung cancer using *Panax notoginseng*.

## Supplementary Information

**Additional file 1.**

## Data Availability

The datasets used and/or analyzed in the present study are available from the corresponding author on reasonable request.

## Abbreviations

CEA: carcinoembryonic antigen
*CTSB*: cathepsin B
EDTA: ethylene diamine tetraacetic acid
ELISA: enzyme-linked immunosorbent assay
IgG: immunoglobulin G
MMP9: matrix metalloprotein-9
NCBI: National Center for Biotechnology Information
NSE: neuron-specific enolase
CYFRA21: Soluble fragment of cytokeratin 19
PCR: polymerase chain reaction
PNS: *Panax notoginoside*
SPF: specific-pathogen-free
TSPN: *Panax notoginoside* treatment
