# Supplementary material for: Mechanism of action of Panax notoginoside against lung cancer in mice based on response to CTSB gene
Source: BMC Complement Med Ther. 2020 Nov 25;20:367. doi: 10.1186/s12906-020-03159-0 (PMC7691060; doi:10.1186/s12906-020-03159-0)
Supplement: Supplementary file 1 — Additional file 1. [file 12906_2020_3159_MOESM1_ESM.docx]

Figure 4

Cathepsin B





GAPDH
